# Designer nucleases to treat malignant cancers driven by viral oncogenes

**DOI:** 10.1186/s12985-021-01488-1

**Published:** 2021-01-13

**Authors:** Tristan A. Scott, Kevin V. Morris

**Affiliations:** Center for Gene Therapy, City of Hope, Beckman Research Institute and Hematological Malignancy and Stem Cell Transplantation Institute at the City of Hope, 1500 E. Duarte Rd, Duarte, CA 91010 USA

**Keywords:** Oncogenic viruses, ZFN, TALEN, CRISPR/Cas, HPV, Gammaherpesvirus, HTLV-1, MCPyV

## Abstract

Viral oncogenic transformation of healthy cells into a malignant state is a well-established phenomenon but took decades from the discovery of tumor-associated viruses to their accepted and established roles in oncogenesis. Viruses cause ~ 15% of know cancers and represents a significant global health burden. Beyond simply causing cellular transformation into a malignant form, a number of these cancers are augmented by a subset of viral factors that significantly enhance the tumor phenotype and, in some cases, are locked in a state of oncogenic addiction, and substantial research has elucidated the mechanisms in these cancers providing a rationale for targeted inactivation of the viral components as a treatment strategy. In many of these virus-associated cancers, the prognosis remains extremely poor, and novel drug approaches are urgently needed. Unlike non-specific small-molecule drug screens or the broad-acting toxic effects of chemo- and radiation therapy, the age of designer nucleases permits a rational approach to inactivating disease-causing targets, allowing for permanent inactivation of viral elements to inhibit tumorigenesis with growing evidence to support their efficacy in this role. Although many challenges remain for the clinical application of designer nucleases towards viral oncogenes; the uniqueness and clear molecular mechanism of these targets, combined with the distinct advantages of specific and permanent inactivation by nucleases, argues for their development as next-generation treatments for this aggressive group of cancers.

## Background

With the advent of genome editing came the notion and promise of next-generation therapeutics. Regardless of modality, designer nucleases function through a convergent mechanism by targeting a specific DNA site through a programmable binding component and then a nuclease component results in a double stranded break (DSB) at the target site forcing activation of repair pathways—non-homologous DNA end joining (NHEJ) or homology directed repair (HDR). Repair through NHEJ is error-prone and, under repetitive cleavage, results in insertion and deletions (indels) causing deleterious frame-shift mutations and gene inactivation, or two nucleases can be used to precisely excise a section of DNA (Reviewed in [1]). HDR relies on a homologous DNA template to accurately repair the target site and through the introduction of an artificial template precise sequence changes to the genome can be made. For the purpose of gene inactivation, NHEJ is the most exploited pathway.

**Fig. 1:**
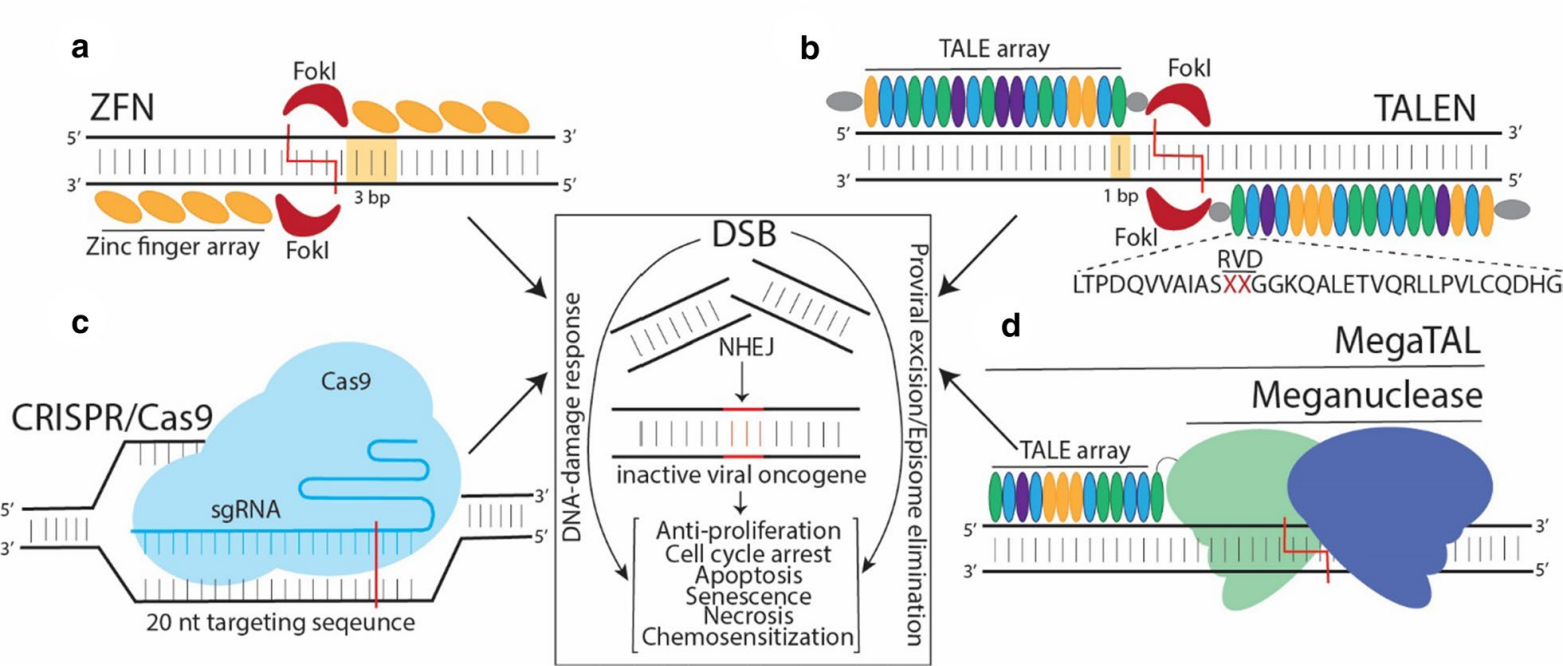
Designer nucleases for inactivating viral oncogenes. Schematics of four different designer nucleases are shown. **a** ZFNs contain individual ZFPs that each bind 3 bp and are combined to form a ZFP array. **b** TALE arrays consist of repeat domains, each binding a unique nucleotide through an RVD (red X). ZFNs and TALENs are generated by fusing a ZFP/TALE to a FokI nuclease. Pairs of effectors bind opposite strands flanking the cleavage site and, through FokI dimerization, results in a DSB (red line). **c** CRISPR/Cas consists of a Cas9 nuclease guided to the target by an sgRNA and through ~ 20 nt complementary to the target DNA, the Cas9 generates a DSB. **d** A meganuclease (homing endonuclease) consists of a heterodimer protein evolved to bind two half-sites, which can be fused to a TALE array (megaTAL) to improve specificity. The DSB in the viral oncogene results in anti-tumor effects either through activation of the NHEJ pathway that introduces deleterious mutations into the viral oncogene, activation of the DNA damage response, or elimination of episomes or proviral excision

There are currently several gene-editing platforms used to target viral oncogenes: zinc finger nucleases (ZFN), transcription activator-like effector nucleases (TALEN), and the clustered regularly interspaced short palindromic repeats with CRISPR-associated protein (CRISPR/Cas) (Fig. 1). Both TALENs and ZFNs exploit customizable DNA binding proteins fused to a FokI nuclease and, as the FokI is a heterodimer, requires two pairs of effectors targeted to opposite strands flanking the target site to reconstitute a functional catalytic FokI to generate a DSB. The binding modules of ZFNs are the Cys2His2 zinc finger proteins (ZFP) that each bind 3 base pairs of sequence, which can be assembled into arrays to recognize longer DNA sequences, although generating ZFN arrays involves complex methodology compared to the more modular assembly of TALENs. The TALEs DNA binding domains were identified as secreted proteins from the *Xanthomonas spp.* bacteria, and consist of highly conversed 33–35 amino acid repeats with two amino acid repeat-variable diresidues (RVD), which dictates individual nucleotide specificity (Fig. 1) [2]. Assembling repeats into TALE arrays flanked by essential TALE-derived N and C-terminal domains fused to FokI repurposes the system for genome editing [3].

CRISPR/Cas was discovered recently as part of the adaptive bacterial immune system and shown to be programmable to unique DNA sites [4], and was rapidly applied to mammalian genome editing [5]. The Cas9 system (class 2, type II) is an RNA-guided endonuclease that consists of a nuclease Cas9 interacting with a trans-activating RNA (tracrRNA) scaffold, which is directed to a target site by ~ 20 nt complementary sequence in the CRISPR RNA (crRNA) flanked by the 3′ protospacer adjacent motif (PAM; *Streptococcus pyogenes* Cas9 (*sp*Cas9), PAM is 5′-NGG-3′), and extensive sgRNA:DNA complementarity results in Cas9 cleavage. The crRNA and tracrRNA can be expressed as a chimeric RNA joined by a tetraloop and referred to as a single/small guide RNA (sgRNA) [6].

The ability to rationally design nucleases against a disease-causing DNA target represents an unprecedented level of precision in the treatment of disease, and designer nucleases have been applied to inactivate integrated and episomal viral DNA genomes, like in the case of human immunodeficiency virus (HIV) [7] and hepatitis B virus (HBV), respectively [8]. Furthermore, nucleases targeting various monogenetic targets are in clinical trials signifying the potential for the clinical translation of designer nucleases (Reviewed in [9, 10]). There are many endogenous and environmental factors that result in the uncontrolled state of proliferation observed in cancer, but the notion that in some cancers a transmissible agent might play a role was paradigm shifting. Approximately 15% of cancers globally are caused by viruses [11]. Why oncogenic viruses result in cancer is a matter of debate as in most cases it’s an evolutionary dead end for the virus, unable to produce progeny as a result of the truncation, integration, or epigenetic suppression of the viral genome. There are postulations that cancer is an unfortunate consequence of immune evasion by the virus, as a result of the overlap in proliferation and innate immunity pathways, which results in oncogenic transformation. This idea is supported by the fact that the majority of oncogenic viral infections are asymptomatic, and only a fraction of persist infections results in malignant transformation, which occurs decades after initial exposure to the infectious agent.

Known etiological viral agents associated with various cancers have been established (Table 1), and these viruses have been implicated not only in tumor formation and clonal expansion, but in the maintenance of the tumor state. These viruses encode ‘oncoproteins’ as well as the more recently explored ‘onco-RNAs’—non-coding RNA or virally derived microRNAs (miRNAs)—that collectively target cellular factors to augment host cell survival, immune evasion, apoptotic pathways, and cell cycle progression for the purposes of viral persistence, but, ultimately, can result in oncogenic transformation. Oncogene addiction, the dependence of a cancer cell on a single gene, or a limited subset of genes, for survival has been described in some virus driven tumors, where tumor maintenance in entirely reliant on the viral oncogenes. However, it must be noted that oncogene addiction has only been formally proven in the context of human papillomavirus (HPV)-related cervical cancers, but there is significant evidence of other virus-associated cancers where the tumor phenotype is augmented by the presence of viral oncogenic elements.

**Table 1:** Oncogenic viruses, associated malignancies and oncogenes

Etiological agent	Malignancies and other disorders	Targeted and potential oncogenes	Review reference
HPV	Cervical cancer Oropharyngeal cancer Vagina cancer Penile cancer Anal cancer Vulvar cancer	E6^a^ E7^a^	[12, 13]
HTLV-1	Adult T-cell leukemia/lymphoma (ATL) HTLV-1 associated myelopathy/tropical spastic paraparesis (HAM/TSP)	HBZ RNA HBZ protein	[14]
EBV (HHV-4)	Burkitt’s lymphoma (BL) Hodgkin’s lymphoma (HL) Diffuse large B cell lymphoma (DLBCL) NK/T cell lymphoma Post-transplant lymphoproliferative disorders (PTLD) Nasopharyngeal carcinoma (NPC) Gastric carcinoma (GC)	Latent coding EBNA1^a^ EBNA2 EBNA3A EBNA3C LMP1^a^ LMP2A^a^ Latent non-coding EBERs EBV miRNAs^a^	[15, 16]
KSHV (HHV-8)	Kaposi's sarcoma (KS) Primary effusion lymphoma (PEL) Multicentric Castleman disease (MCD)	Latent coding LANA^a^ Kaposin Viral cyclin (vCyclin) Viral FLIP (vFLIP) Latent non-coding K12-miRNAs^a^	[17, 18]
MCPyV	Merkel cell carcinoma (MCC)	Large antigen (LT)^a^ Small antigen (sT)^a^	[19]

Etiological agents with a list of their known associated cancers and other related disorders are shown. The genes that are the main oncogenic drivers of these cancers are highlighted as well as other potential targets for nuclease inactivation

^a^Studies inactivating the oncogene with designer nucleases

This concept creates a basis for these viral oncogenes to be targets of therapeutic intervention and, specifically, targeted nucleases. Many oncoproteins are non-enzymatic making them ‘undruggable’, a feature immaterial for designer nuclease applications. Furthermore, targeted nucleases have a distinct advantage over other gene knockdown strategies like RNA interference (RNAi) or antisense oligonucleotides (ASO) as they result in permanent inactivation of the oncogene. This review explores potential oncogenic targets in virus-associated cancers, current nuclease modalities to these sites and potential alternative targets, as well as the challenges and considerations for the therapeutic development of designer nucleases.

## Human papillomavirus

By far the oncogenic virus with the most developed platform of targeted nucleases is HPV, since a third of viral-associated cancers are caused by HPV [20]. As the causative agent of cervical cancer, the second most common cancer in woman and a leading cause of death in developing countries, it is responsible for approximately ~ 450,000 new cases and 233,000 deaths per year with a low 5-year survival rate in the advanced stages of disease [21]. HPV is detected in over 95% of cervical cancer biopsies, a clear association, but there are many high-risk subtypes of HPV involved in other cancers such as anal and oropharyngeal cancers (Table 1) (Reviewed in [12]). Vaccination against high-risk subtypes is an effective strategy to prevent HPV-related cancers, but the high cost of the vaccine, low vaccine coverage in some countries, and the lack of anti-cancer effects within infected individuals suggests there will be HPV-related cancers in the foreseeable future, which urges the development of novel therapeutics.

### E6 and E7

HPV is a non-enveloped virus with a ~ 8 kb circular dsDNA genome, that infects the basal layer of the epithelium. Most individuals clear HPV within 2 years but if not cleared results in long-term persistence, a key feature in HPV-induced cancers [22]. Most HPV subgroups do not cause cancer, but the alpha papillomaviruses are associated with malignancies with the most common cancer-associated subtype being HPV16 followed by HPV18. The transformation of healthy cells to a tumor state has been extensively studied, and it is well understood that accessory proteins E6 and E7 are involved. The E6 protein causes the degradation of the tumor suppressor p53 and actives a common cancer-associated enzyme, telomerase, while E7 degrades the Retinoblastoma protein (pRb) releasing the E2F family of transcription factors to active various downstream genes involved in cell cycle progression [13]. Integration of HPV is also an essential event in oncogenesis, a terminal event for HPV propagation, and normally occurs at fragile sites where the HPV episome is tethered to the host genome [23], inserting the long control region (LCR) promoter with the E6 and E7 genes. Out of the episomal context, E6/E7 expression increases resulting from the loss of LCR regulation by the viral E2 regulatory protein, and increased cellular proliferation, inactivation of cell-cycle check point inhibitors, genomic instability, and accumulative mutations in host genes results in cancer progression. Verification that these proteins are oncogenic drivers and possible therapeutic targets is that E6 and E7 together, or E7 alone, immortalize primary epithelial cells [24], causes epidermal and mucosa hyperplasia in transgenic mouse models [25], and gene knockdown results in inhibition of tumor growth in vitro and in vivo [26].

### Designer nucleases to HPV

The first demonstration of CRISPR/Cas inhibiting HPV-related cancers was sgRNAs targeted to HPV16′s E6 and E7 proteins in the cervical cancer-derived SiHa cells, which increased p53 and it downstream factor, p21, resulting in reduced cell viability [27]. Furthermore, cells transfected with an anti-HPV CRISPR vector transplanted into mice reduced tumor volume and size. The authors also designed a sgRNA to the LCR promoter, which inhibited both E6 and E7 expression and enhanced tumor volume reduction in vivo, representing a compact approach to inhibit both genes through promotor inactivation. Anti-tumor CRISPR/Cas activity against other subtypes was explored through targeting E6 and E7 in the HPV18-transformed Hela cell line resulting in reduced viability through activation of p53 and, important to this study, pRb upregulation that caused cell cycle arrest [28], verifying that both high-risk subtypes could be inhibited using targeted nucleases.

HPV cell lines have an irregular number of integrated copies from 1–2 copies/cell in SiHa cells to ~ 500 copies/cell in CaSki cells [29], which may be an issue for effective target inactivation. However, targeting the HPV16 E7 gene showed similar induction of apoptosis, reduction in viability and pRb activation in both cell lines [30], which suggests integrated copy number is not a factor. As it is unlikely that all copies in CaSki cells were inactivated, DSB may be sufficient to active proapoptotic pathways, and anti-tumor effects are not strictly limited to oncogene mutation, which has been suggested by others [28] (Fig. 1). Also, this suggests that even a momentary reversal of the “oncogenic amnesia” perpetrated by the viral oncogenes [31]—a model purposing that the tumor cell is oblivious to its state of genomic insult—is enough to restore checkpoint inhibition and halt tumorigenesis. However, the contribution of individual integrations to tumorigenesis in CaSki cells has not been determined, and only a subset may need to be inactivated, which could also explain the similar inhibitory profiles.

The above studies represent cervical cancer cell lines, but HPV causes a range of malignancies (Table 1). Whether other HPV-related cancers could also be inhibited by targeted nucleases was explored using CRISPR/Cas inactivating an HPV18 E6 gene in an oral squamous cell carcinoma, HSC-2, and resulted in reduced proliferation with increased apoptosis [32]. Targeting of HPV16 in an oropharyngeal squamous cell carcinoma, SCC2, was explored, but inactivation of E7 alone was insufficient to affect cell survival and required CRISPR targeting of both E6 and E7 to reduce viability by ~ 50% [33]. Other studies targeted E7 in other subtypes, HPV6 and 11, which are low-risk wart causing cancers in primary keratinocytes and observed reduced viability [34]. These studies provide evidence that targeted nucleases could be generally applied to inhibit HPV-induced tumors.

Delivery remains a significant hurdle for any gene therapy, and programmable nucleases are no exception. Various viral vectors can be used to deliver and express transgenes, but a commonly exploited vector is the adeno-associated viral vector (AAV). AAV-2 was used to deliver a E6 sgRNA, albeit to HPV cell lines overexpressing Cas9, and when administrated intratumorally to subcutaneously implanted tumors in mice, it reduced tumor size and volume [32]. Although viral vectors are a promising vector for gene therapy, limitations in targeting viral oncogene with designer nucleases exist, which is discussed below (see Delivery).

Promising non-viral vector approaches have been explored to deliver anti-HPV designer nucleases. Early stage HPV cervical intraepithelial neoplasia 1 or 2 (CIN1/2) are usually treated by cauterization or excision, but these methods are associated with increased risk of miscarriage and premature birth. The HPV neoplasm can return, and recurrent HPV is associated with a higher risk of cervical cancer. Another delivery approach focused on local vaginal administration to inactivate early stage HPV cancers. Hu et al. [35], developed a series of paired TALENs to target E6 and E7 genes in HPV16 and 18. Optimized through N-terminal domain truncations and FokI mutations, the best TALENs resulted in increased apoptosis and reduced cell growth in vitro and tumor inhibition in transfected tumors in vivo. Furthermore, the HPV16 E7 TALENs were delivered using a cationic polymer intravaginally to K14-HPV16 transgenic mice. Local delivery almost completely inactivated the E7 protein with a concordant reduction in hyperplasia and reversal of the malignant phenotype in the cervix. Furthermore, proliferation markers were reduced with restoration of pRb levels and cell cycle regulatory factors. No lymphocyte infiltration resulted from TALEN treatment suggesting no immune response to the bacterially derived TALE components, and collectively this provides strong support for a minimally invasive and effective means to inactive early stage HPV cervical cancers. Ding et al. [36], verified ZFNs as an anti-HPV tumor modality targeting E7 in HPV16 and 18. The ZFN were bound to a cationic polymer and injected into HPV-associated tumors, which increased apoptosis markers while reducing tumor size and proliferation markers, and likewise could be used in local and topical applications. The cationic polymer represents a means for local delivery of designer nucleases but is not likely viable for systemic administration.

One of the most developed delivery systems was a recent study by Jubair et al. [37], who used a Cas9 and E7-sgRNA vector loaded into ‘stealth’ liposomes. These are lipid nanoparticles that are PEGylated to prevent opsonization, increase circulation time and stability while reducing toxicity. Systemic delivery of the CRISPR components to the tumor occurred through ‘passive targeting’ resulting from higher retention in tumors from increased angiogenesis and permeability. Administrated intravenously, inhibition of subcutaneous tumor volume was observed with increased apoptosis and mouse survival. Impressively, the remaining benign nodule was negative for p16, an upregulated marker in HPV-tumors, suggesting effective eradication of the oncogenic drivers.

Purified protein may also be a form in which to prepare therapeutic recombinant nucleases to inactive HPV. Novel HPV promoter targeting modalities have been developed using the bovine HPV E2 fused to FokI (BEF) [38], but this system suffered from poor cleavage and mostly suppressed the promoter through E2 protein binding. However, expanding upon this concept, Miro et al. [39], developed an artificial zinc finger (AZP), a single ZFP targeted to the E2 binding site in the HPV 18 promoter, which was fused to either a Staphylococcal nuclease (SNase) [40], or an optimized single-chain FokI dimer (scFokI) [41, 42], and this compact nuclease platform is capable of generating DSBs without the need for two ZFN effectors. Furthermore, ZFPs are intrinsically cell permeable proteins [43] and when fused to cell penetrating peptides (CPP) [44, 45] could facilitate direct internalization of the recombinant nuclease. When the CPP-modified AZP-SNase was applied directly to cells it was able to inhibit an HPV replication vector [40, 46]. Unfortunately, the authors did not show anti-tumor effects in any HPV-transformed cell lines, but nevertheless it is conceivable that direct application of cell-penetrating recombinant protein could also be applied for the local treatment of HPV.

Inactivating viral oncogenes can result in anti-tumors effects through apoptosis, necrosis, or senescence. A preprint manuscript that targeted HPV18 E6 and E7 genes with CRISPR/Cas explored the anti-tumor mechanisms [47], and, contrary to other studies, apoptotic features were no present but instead markers of senescence were observed. Shankar et al. [48, 49], tested TALENs to HPV16 E7, and observed a distinct lack of apoptosis, but instead features of cell cycle arrest and necrosis. Collectively, these studies show that a pleiotropic route to tumor inhibition occurs upon oncogene inactivation in HPV-related cancers.

## Human T-cell leukemia virus type 1

Human T-cell leukemia virus type 1 (HTLV-1) is a retrovirus that has a broad global distribution and has infected ~ 10 million people through sexual contact, mother-to-child transmission, or blood exposure of which 2–5% will develop malignancies [14]. It forms a life-long infection within individuals through proviral integration into the host genome with a notable tropism for its primary cell of transformation, CD4^+^ T-cells. Although most people will be asymptomatic, after a long latency period (> 30 years) HTLV-1 infection can result in a devastating cancer, adult T-cell leukemia/lymphoma (ATL), where in its most common form of acute ATL the median survival from diagnosis is ~ 9 months even with aggressive treatment [50], urging the development of alternative therapeutic strategies.

### HBZ

The ~ 9 kb HTLV-1 proviral genome is flanked by 5′ and 3′ long terminal repeat (LTR) promoters, which can transcribe notable non-structural oncogenes from the 5′ and 3′ LTRs, the Tax and HTLV-1 bZIP factor (HBZ) genes, respectively. Transformation results from a complex interplay between these two proteins, and Tax’s transformative contributions to ATL are extensively studied (Reviewed in [14]). Although undoubtedly important in oncogenic transformation, Tax is highly immunogenic resulting in immune clearance and, in ALT, Tax is almost always inactivated through truncation, mutation or epigenetic suppression of the 5′ LTR [51, 52], questioning its role as ATL’s main oncogenic driver.

The discovery of the anti-sense HBZ transcript [53] has sparked elucidation of its role in oncogenic transformation, maintenance as well as pathological features of the disease. The non-immunogenic HBZ is expressed in all ATL patient-derived tumors [54, 55] and activates pro-survival genes [56], supports proliferation [57–59], upregulates C–C chemokine receptor 4 (CCR4), a distinct marker of ATL that augments ATL migration and proliferation [60], causes bone degeneration through RANKL/*c-Fos* pathways [61], is anti-apoptotic [62], upregulates telomerase [63], and promotes genomic instability through cellular miRNAs [64]. Importantly, transgenic mice expressing HBZ develop T-cell malignancies [65], and inhibition of HBZ in HTLV-1 transformed cell lines reduces proliferation [66], highlighting HBZ as a potential oncogenic target in ATL for nuclease-mediated inactivation. Interestingly, both the HBZ mRNA and protein have distinct functions [56], suggesting that inhibiting both the protein and RNA may be required to augment HBZ-mediated effects.

### Designer nucleases to HTLV-1

Currently, only one study has targeted the LTR using ZFNs, which reduced proliferation in various HTLV-transformed and patient-derived ATL cell lines, and reduced tumor volume in mice [67]. The HBZ-specific effects of ZFNs were not investigated, and inhibition could result from several possible mechanisms, (1) mutation of transcriptional regulatory elements in the LTR affecting HBZ expression, (2) excision of the proviral genome, including HBZ, by cleaving the flanking LTRs, or (3) apoptosis through activation of DNA damage responses (Fig. 1). A more focused strategy could target the early HBZ coding sequence as the RNA structure that supports proliferation has been elucidated [57], resulting in inactivation of both the RNA and protein functions of HBZ. With a clear molecular target and demonstrated efficacy, further development of targeted nucleases to HTLV-1 should be considered as a treatment strategy for ATL.

## Gammaherpesviruses

The *Gammaherpesvirinae* subfamily includes the first human oncovirus discovered, Epstein–Barr virus or human herpesvirus-4 (EBV/HHV-4), and Kaposi’s sarcoma-associated herpesvirus (KSHV/HHV-8), which are membraned viruses with large dsDNA genomes of ~ 170 kb. EBV is a common, persistent infection present in ~ 95% of humans and results in ~ 1% of all cancers consisting of lymphoid and epithelial malignancies, the most common being B-cell lymphomas and nasopharyngeal carcinoma (NPC) (Table 1). KSHV infection occurs at a young age in endemic areas or transmitted by oral shedding and bodily fluids, but rarely causes disease in healthy individuals. KSHV malignancies generally occur in immunocompromised individuals epidemiologically linked to the early days of the AIDS pandemic, or in induced immune suppression such as an organ transplant. It infects a wide range of epithelial and immune cells and is the etiological agent of the epithelial-derived Kaposi’s sarcoma (KS) (Table 1), and other lymphoproliferative disorders, like primary effusion lymphoma (PEL), a rare neoplasm with a median survival of 1 year. Although incidences of KS have reduced with antiretroviral therapy (ART), half of the AIDS-related KS cases never achieve remission with no known cure [68], and is associated with significant mortality in developing countries [69].

### Latency-associated genes

EBV and KSHV have multicopy circular genomes present in the nucleus, which rarely integrates, and exists as episomes tethered to, and copied with, the host DNA. Herpesviruses have lytic and latent states, but oncogenesis is generally associated with latency. The latent genome encodes a range of latency coding and non-coding RNAs that play a role in immune evasion, enhance survival and cycle cell progression, manipulate cell signaling as well as control viral latency (Table 1) [15]. Unlike the relativity few oncogenes in other virus-associated cancers, herpesviruses have a concert of elements that contribute to episome stability and oncogenesis, which could be noteworthy targets for designer nucleases.

### Designer nucleases to EBV and KSHV

The oncogenic ability of EBV is clearly established as it can transform its main cell type, B lymphocytes, in vitro [70], and the presence of EBV provides a survival advantage to the tumor cell [71]. The EBV genome encodes a wide range of factors that promote tumorigenesis, so strategies have focused on the eradication of the EBV genome through either nuclease-mediated cleavage and degradation, or targeting viral proteins involved in episome maintenance. After EBV infection, the genome progresses through a series of latency programs with an ever-reducing number of expressed genes, but EBV nuclear antigen 1 (EBNA1) is common to all latency states. Apart from its roles in immune evasion and oncogenesis [72], EBNA1 is essential for the persistence of the viral genome as inhibitors reduce the genome copy number [73] and EBNA1 depletion inhibits tumor proliferation [74–76], making it a rational anti-tumor target.

However, the anti-proliferative effects of inactivating EBNA1 or genomic depletion in several studies has either been difficult to achieve, or not characterized for anti-tumor effects. Noh et al. [77], targeted EBNA1 with TALENs, which required multiple treatments and clonal expansion to observe reduced proliferation in the edited clones. Nevertheless, reduced clonal outgrowth was observed in EBNA1 ‘low’ clones in two lymphocyte and one gastric tumor cell lines, demonstrating proof-of-concept that inactivating EBNA1 with nucleases could suppress a range of EBV malignancies. Wang and Quake [78], used CRISPR/Cas with seven sgRNAs targeted to numerous repeat sequences simultaneously in order to increase the likelihood of episome fragmentation. Large deletions were observed in enriched cell populations resulting in an induction of apoptosis and an 85% reduction in viral genome copy number. Others have also confirmed the reduction in EBV copy number when targeting EBNA1 and oriP (the EBNA1 binding site in the episome), and a combination of sgRNAs were needed for high-level episome eradication (up to 95%), but anti-tumor effects were not studied [79]. Another study targeting EBNA1, oriP and W repeats with multiple sgRNAs in NPCs reduced EBV DNA up to 50%, but did not reduce viability, which might reflect issues with observing effects in bulk cell populations and an episome ‘eradication threshold’ for anti-tumor effects [80]. In KSHV, latency-associated nuclear antigen (LANA) is likewise involved in episome maintenance, suppresses p53 [81] and pRb [82], and its knockdown resulted in KSHV episome reduction and apoptosis in PEL cells [83]. CRISPR/Cas was tested against LANA and delivered using a replication-incompetent adenovirus type 5 (Ad5) as a viral delivery platform [84]. Immortalized cell lines subsequently infected with KSHV were treated with the Ad5-CRISPR vector, which reduced LANA protein and nearly eradicated the episomes. As the cell lines were not KSHV-dependent, effects on growth could not be observed, but may be a promising approach to deplete KSHV genomes. The loss of genomes was also observed when engineering CRISPR knockouts of the ORF57 gene in PEL cells [85], which further suggests that simply targeting the episome could result in nuclease-mediated degradation (Fig. 1). Nevertheless, virus genome depletion as an anti-tumor strategy in KSHV and EBV requires significantly more characterization as it’s unclear if this represent a viable approach.

Both EBV and KSHV tumors have numerous episome copies but, unlike in HPV-associated tumors, the copy number may be a hurdle for complete herpesvirus eradication. Inactivating essential oncogenes may better mitigate the malignant phenotype in the event of incomplete genomic elimination. Latent membrane protein 1 (LMP1) is an established oncogene [15] and, if inhibited, results in cell cycle arrest [86]. Overexpressing LMP1 promoted cell growth that was inhibited by anti-LMP1 sgRNAs [87], suggesting this could happen in the context of an EBV-dependent tumor and should be explored further as a target. In KSHV, viral FLIP (vFLIP) and viral cyclin (vcyclin) genes are part of the KSHV oncogenic latency cluster [88] (Table 1), and knockdown reduced PEL tumor growth in vitro and in vivo [83], highlighting these genes as possible targets for future studies. Alternatively, the majority of PELs are co-infected with EBV and EBNA1 inhibition reduced KSHV-dependent cell line proliferation [89], opening up the possibility of combined therapeutics for EBV and KSHV in dually infected cells.

In leu of protein coding genes, a variety of non-coding RNAs are expressed from the latent herpesvirus episome. MiRNAs are small non-coding RNAs that regulate target genes through RNA silencing, and KSHV encodes an important K12 cluster of miRNAs (K12-miR) involved in tumorigenesis, immune evasion, and maintenance of latency (Reviewed in [17]). Liang et al. [90], targeted and excised the miRNA promoter in PEL cell lines and showed that the miRNA levels were reduced with a concordant increase in viral lytic genes. Notably, treated cells proliferated slower and reversed the inhibitory effects of the miRNAs on their cellular cell cycle and signaling targets. EBV has a multitude of viral miRNAs expressed during latency that are involved in oncogenesis (Reviewed in [16]), and inactivation of EBV miRNAs inhibited cell growth in vitro [91]. CRISPR sgRNAs that excise the EBV miRNA promoter have been shown to mitigate the miRNA’s inhibition of their cognate targets [79], and should be explored further to inhibit EBV-dependent proliferation, which may serve as more rational anti-tumor target than ‘brute force’ genomic eradication.

There are significant challenges for targeting herpesvirus-associated malignancies with designer nucleases. Current targets lack efficacy in bulk populations or anti-proliferative effects in relevant cell models. Importantly, there are no in vivo studies confirming the anti-tumor effects of targeted nucleases for these herpesvirus-associated cancers*.* Furthermore, whether the anti-proliferative effects observed in KSHV PEL cells will translate into KS or multicentric Castleman disease (MCD) remains to be determined, but the lack of KS cell lines is a hindrance. As mentioned, there are a large number of episomes that would need to be effectively eradicated (up to 800 copies in some NPCs [92]), and the extent of genome eradication needed to get an inhibitory effect is currently unknown and strongly urges further characterization, or the identification of more discrete anti-tumor targets.

## Merkel cell polyomavirus

Merkel cell polyomavirus (MCPyV) is the most recently discovered oncogenic virus [93] and is ubiquitously present in the human population as it is acquired in the first years of life, and generally does not cause any symptoms. Polyomaviruses have long been known to transform cultured cells (SV40) [94], but it was many years before MCPyV, the first human oncogenic polyomavirus agent, was found in 80% of Merkel cell carcinomas (MCC), a rare and aggressive skin cancer [93]. MCC occurs in immunocompromised individuals, but mostly in the elderly with a median diagnosis age of ~ 70 years old [95], but with a 95% increase in MCC diagnoses since 2000 [96] and a 5-year mortality rate of > 40%, makes it an aggressive skin cancer in need of therapeutic solutions.

### LT and sT

The reason for oncogenic transformation is still being elucidated but, like HPV, integration of MCPyV is required. MCPyV is a non-enveloped, circular dsDNA virus about ~ 4 kb and in MCC there is the monoclonal integration of the tumor antigen (TA) open reading frame, which expresses the large (LT), small (sT), 57kt, and ALTO transcripts [97]. Truncation of the LT is required, which importantly disables viral replication domains, but retains pRb interactions for cell cycle regulation; both features needed for oncogenesis [98, 99]. The oncogenic involvement of the 57kt and ALTO proteins are not well understood, but sT, a part from increasing LT expression [100], promotes oncogenesis through cap-dependent translation [101], functionally inhibits p53 [102], and is a main oncogenic driver as it alone can transform rat fibroblasts [101], co-operates with LT to promote growth in human fibroblasts [98], and generates carcinomas in transgenic mice [103]. Unlike LT, sT is present in all MCPyV-positive MCCs [101], but even though sT knockdown has similar growth inhibition to a combined suppression of LT and sT, its reduction alone did not affect cell cycle progression or cause apoptosis [101], suggesting inhibition of both oncogenes would be beneficial for anti-tumor effects. Furthermore, knockdown studies have shown that MCCs are TA-dependent in vitro [104] and in vivo [105] verifying these oncogenes as possible targets for nuclease-mediated inactivation.

### Designer nucleases to MCPyV

CRISPR/Cas sgRNAs targeting the LT or a common sT/LT site were tested in a patient-derived MCC cell line, which resulted in reduced proliferation with cell cycle arrest, apoptosis, and necrosis [106]. In exploring the mechanism in more detail apoptotic markers were not present, but the reestablishment of various cell cycle regulatory features was observed, which has been noted by others [104], and likely a result from reactivation of the pRb pathway [105]. These results are promising and future work should confirm these effects within in vivo models.

## Targeted nucleases and chemosensitization

Viral oncogenic drivers can offer pro-survival advantages to tumor cells, which can diminish the effects of chemotherapy. ATL is refractory to conventional chemotherapy and HBZ activates the brain-derived neurotrophic factor/TrkB signaling pathway, a pro-survival pathway implicated in chemoresistance [107]. Furthermore, Survivin, an inhibitor of apoptosis and also associated with chemoresistance, is upregulated by many of the mentioned viral oncoproteins [56, 108−110]. This chemosensitization through nuclease-mediated inactivation of viral oncogenes was demonstrated when Zhen et al. [111], showed in vitro and in vivo that CRISPR/Cas9 targeting of HPV E6 and E7 improved the toxic effects of cisplatin and EBV-sgRNAs to EBNA1 increased tumor sensitivity to cisplatin and fluorouracil [80], suggesting targeting viral oncogenes could improve responses to traditional chemotherapeutic agents.

## Nuclease escape and oncogenic virus variation

Evolving targets, like viruses, can escape targeted treatment. However, the replicating factors of oncogenic viruses are either epigenetically suppressed (EBV/KSHV) or lost due to integration and inactivation (HPV, HTLV-1, MCPyV), and so the likelihood of virus-induced escape is relatively low. Variation within the genomically unstable cancer cell may also select for escape mutants, but as a result of the clonal expansion of many virally-driven tumors, this may not be an issue [14, 93, 112]. “Nuclease-mediated escape” results from the introduction of NHEJ mutations that are tolerated within the oncogenes (silent mutations/in-frame deletions), but prevents nuclease re-cleavage, which has been observed in CRISPR/Cas9 targeting of HIV [113] as well as herpesviruses [79]. It must be noted in the case of HPV, tumor suppression improved with repetitive doses in vivo resulting in complete oncogene eradication arguing against this type of escape in the context of HPV [37]. Nevertheless, this escape arises in type II Cas9 systems because the cleavage site is over the crucial binding nucleotides in the sgRNA, the ‘seed’ sequence, which is intolerant of the introduced mutations. “Nuclease-mediated escape” can be avoided by using multiple sgRNAs [79, 114], or by exploring other CRISPR types. In the type V Cas12 system, the ‘seed’ sequence is distal to the cleavage site and the crRNA is more tolerant of the introduced mutations favoring re-cleavage and ensuring target site inactivation, a feature leveraged to great effect in the complete inhibition of HIV escape mutants with a single Cas12 crRNA [115]. Of note, this escape may not be an issue for ZFNs or TALENs as their binding sites flank the cleavage site, but this eventuality remains to be assessed.

Oncogenic viruses have a wide range of strains and subtypes and, in some cases, significant sequence variation. Some viruses, like HTLV-1, are highly conserved even amongst geographically distant subtypes [116], but the HPV E6 and E7 genes are poorly conserved making each targeted nuclease HPV subtype-specific (Reviewed in [12]). This variability may necessitate multiple targeted nucleases to effectively treat a broad range of viral subtypes. The easily programmable nature of CRISPR/Cas technology may be better suited for multiplex targeting, where pools of Cas9 sgRNAs could be utilized. Alternatively, exploiting features of the Cas12 CRISPR system, which uses compact arrays of pre-crRNAs processed by the Cas12 enzyme into a series of mature crRNAs [117], is potentially capable of targeting multiple oncogenic subtypes simultaneously. Nevertheless, targeting highly conserved regions should be an upfront factor in nuclease design to ensure its broader therapeutic applicability.

## Specificity

Specificity of the targeted nucleases is paramount as off-target DSBs in the genome of healthy cells could result in tissue and organ damage or even cancer, and many of the mentioned studies were lacking in considerations towards specificity (Table 2). The topic of designer nuclease specificity has been covered extensively (Reviewed in [118, 119]), and so, briefly, as a result of the rational design of targeted nucleases, off-targeting can be mitigated, or at least thoroughly quantified, for the following reasons: (1) TALENs and ZFNs use an obligate FokI heterodimer and this ‘two-part’ requirement to generate DSBs has built-in specificity, (2) designer nucleases can be bioinformatically screened for low off-target profiles within the human genome, (3) Cas9 specificity can be improved by using rationally engineered or evolved high-fidelity Cas9 mutants, or, similar to the ‘two-part’ recognition of the ZFN/TALEN systems, use a nickase Cas9 or a catalytically dead Cas9 fused to FokI that requires two adjacent sgRNAs to generate DSBs, (4) truncation of the sgRNA (tru-sgRNA) can improve its specificity by removing nonessential nucleotides, (5) using the nucleases in more transient forms such as mRNA or a ribonucleoprotein (RNP) will prevent long-term persistence of the nuclease, which is associated with higher off-target effects, (6) the off-target profile of designer nucleases can be objectively quantified and qualified through an extensive array of unbiased assays in vitro and in vivo, and (7) the viral oncogene represents a unique target that is not present in the human genome. Nevertheless, targeted nucleases towards oncogenic viruses should be thoroughly vetted prior to therapeutic applications.

**Table 2:** Targeted nucleases to oncogenic viruses

Viral target	Modality	Target	Cell lines	In vivo	Vector	Delivery method	Reference
HPV	*sp*Cas9 or d*sp*Cas9-FokI	E6, E7, LCR	SiHa	Yes	Plasmid DNA	No	[27, 111]
		E6, E7	Hela, SiHa	No	Plasmid DNA	No	[28]
		E7	SiHa, CaSki	No	Plasmid DNA	No	[30]
		E6	Hela, HSC-2, SKG-I	Yes	AAV genome	AAV-2^a^ (intratumoral)	[32]
		E6, E7	Hela, CaSki	Yes	Plasmid DNA	‘Stealth’ Liposomes (Intravenous)	[37]
		E6, E7	Hela	No	Plasmid DNA	No	[47]
		E6, E7	SCC2	No	Plasmid DNA	No	[33]
	TALEN	E6, E7	SiHa, S12, Hela	Yes	Plasmid DNA	Cationic polymer (Topical)	[35]
		E7	SiHa, CaSki	No	Plasmid DNA	No	[48, 49]
	ZFN	E7	SiHa, Hela, CaSki, S12	Yes	Plasmid DNA	Cationic polymer (Intratumoral)	[36]
	BEF	LCR	Hela	No	Plasmid DNA	No	[38]
	AZP-SNase or scFokI	LCR	None	No	Plasmid DNA, Protein	No	[39 − 42, 46]
HTLV-1	ZFN	LTR	C8166, S1T, ED	Yes	Plasmid DNA	No	[67]
EBV (HHV-4)	*sp*Cas9	EBNA-LP, 125 bp repeat, EBNA3C, EBNA1, pstI repeat, LMP1	Raji	No	Plasmid DNA	No	[78]
		EBNA1, oriP, miRNA promoter	SNU-719	No	Lentiviral vector	No	[79]
		LMP1	EBV infected cell line	No	Plasmid DNA	No	[87]
		EBNA1, oriP and W repeats	C666-1	No	Plasmid DNA	No	[80]
	TALEN	EBNA1	Raji, SNU-719, SNU-265	No	Plasmid DNA	No	[77]
KSHV (HHV-8)	*sp*Cas9	LANA	Vero219, L1T2, BC3	No	Adenoviral genome	Replication-deficient Ad5 (Intratumoral)	[84]
		K12-miR promoter miR-K12-1 miR-K12-9	BCP-1, BCBL-1	No	Plasmid DNA	No	[90]
MCPyV	*sp*Cas9	LT and sT	MS-1, WaGa	No	Plasmid DNA	No	[106]

The modalities that have been developed against their respective oncogenic viruses and targets in each study are shown. The in vitro cell lines as well as whether in vivo studies were performed are highlighted. The delivery vectors developed are indicated along with the route of administration used in vivo. The expression vector used for the different nuclease modalities are indicated

^a^AAV delivered gRNA to Cas9 overexpressing cell line

## Delivery

Delivery of designer nucleases to tumor cells remains a significant challenge for its therapeutic application. The type of delivery system used will be dictated by the cancer type, stage of cancer, whether local or metastatic, and the involvement of secondary tissues. Careful consideration would need to be given to the selection of a delivery system that best suits the malignancy. Currently, delivery approaches are underdeveloped (Table 2). Two studies used viral delivery vectors, AAVs [32] and Ad5 [84], which may be compatible with local delivery. However, viral vectors have several limitations: (1) The repeat sequences present in ZFPs and TALEs make the engineering and production of viral vectors significantly more difficult, (2) the *sp*Cas9 systems preclude the use of AAVs due to the limited packaging capacity of this vector (~ 4.4 kb for double-stranded AAVs), but could be used with smaller orthologues like *Staphylococcus aureus* Cas9 (*sa*Cas9) [120], *Campylobacter jejuni* (*cj*Cas9) [121], or the smallest Cas variant discovered in ‘huge’ phages Cas12φ [122], (3) the long-term expression of nucleases in tissues other than the tumor is not desirable potentially increasing off-targeting in these tissues or may elicit cytotoxic cellular responses to the bacterial transgene components, and (4) antibody responses to the viral vector would preclude repeat administration. Overall, these limitations suggest non-viral systems may be more applicable for the delivery of targeted nucleases to virally driven cancers.

As discussed, cationic polymers [35] or cell-penetrating proteins [46] could work well for local injection into accessible solid tumors, but unlikely to be compatible with systemic delivery. Nevertheless, these are very promising for topical administration in early stage HPV cervical cancers, but other HPV-affected tissues, like esophageal regions, may not be readily accessible. Furthermore, late stage metastatic HPV would ideally require systemic delivery with a potential viable approach using ‘stealth’ lipid nanoparticles [37], but whether systemic treatment with these particles will reach all secondary sites of HPV metastasis (lymph, lung, liver, and bone marrow) remains to be determined. Furthermore, whether the ‘passive targeting’ of the lipid nanoparticles would effectively deliver nucleases to tumor cells in other virus-associated cancers, especially blood cancers like ATL or B-cell lymphomas, should be assessed. Importantly, plasmid DNA components were used in the majority of the discussed studies (Table 2), which may be problematic as it would express nucleases for extended periods in healthy tissue. Substituting the DNA vectors for transient components combined with lipid nanoparticles, like mRNA [123] or RNPs [124] may improve the ‘druggability’ of therapeutic nucleases. Lastly, CRISPR/Cas9, ZFNs, and TALENs require two components and a single module nuclease may have advantages to simplify economic scale-up and delivery, and the compact high-fidelity meganucleases should be considered for targeted inactivation of viral oncogenes (Fig. 1) [125].

## A note on HBV and HCV

HBV and hepatitis C virus (HCV) are carcinogenic viruses known to increase the risk of hepatocellular carcinoma (HCC), a devastating liver cancer. However, there is no evidence that antivirals will inhibit HCC growth, but rather eliminating these viruses before cancer development may reduce cancer risk [126, 127]. As viral targeting will likely not augment tumor progression, these viruses are beyond the scope of this review. However, there are extensive reviews covering innovative targeted nucleases to HBV and HCV [128, 129].

## ‘Hit-and-run’ virally driven cancers

As described, there are established examples of viral oncogenes contributing to tumor maintenance, pathological features, and chemoresistance and, in the context of HPV-related cervical cancers, an established state of oncogene addiction. However, it must be mentioned that there are examples where the viral components may become dispensable to the tumor phenotype. There are recent postulations for a ‘hit-and-run’ hypothesis suggesting that the accumulation of other genomic mutations could supersede the tumor’s dependence on the viral oncogenes resulting in a virus-independent tumor (Reviewed in [130]). This virus-independence has been observed with the spontaneous loss of EBV episomes in a Burkitt’s lymphoma (BL) cell line [131], or KSHV transformed cell lines that are episome negative but still tumorigenic in mice [132], and even in instances of HPV-related cancers, often considered a clear example of oncogenic addiction, known HPV18 transformed cell lines can lack HPV DNA [133]. Future studies should focus on clarifying the context and extent to which these cancers are dependent on viral elements, as this insight would allow for the cognizant application of nucleases in the treatment of virus-associated cancers. Furthermore, in situations where the viral oncogenes are completely lost and go undetected in the tumor biopsies, a ‘hit-and-run’ hypothesis may suggest a larger contribution of oncogenic viruses to diagnosed cancers than previously thought, which could provide a rationale for the early detection and prophylactic targeted inactivation of high-risk viral oncogenes to prevent cancer.

## Conclusion

Oncogenic viruses are a global health crisis, often aggressive with poor responses to current treatments, necessitating the development of novel therapeutics (Table 2). The potential for targeted nucleases to inhibit virally driven cancers cannot be overstated as unlike many non-viral cancers these offer a critical and unique oncogene that is foreign, and are ideal targets for designer nuclease-mediated inactivation, which could permanently inactivate tumorigenic drivers while improving responses to standard chemotherapeutic regimens. Encouragingly, there are registered clinical trials using anti-HPV ZFNs, TALENs and CRISPR/Cas for local vaginal treatment (Reviewed in [10]) and, if positive effects are observed, would be a significant alternative approach to treat recurrent HPV, and provide strong support for the future development of these modalities with appropriate delivery vectors against late-stage metastatic HPV. Although there are a number of studies validating the anti-tumor effects of targeted nucleases to viral oncogenes, there is considerable challenges before these can be applied broadly in clinic such as the validation of effective anti-tumor viral targets in representative cell line models, observing anti-tumor effects in vivo in combination with viable delivery platforms, and the assessment of safety profiles. Nevertheless, designer nucleases offer a novel treatment approach that could be transformative for patients suffering for aggressive virally driven malignancies.

## Data Availability

Not applicable.

## Abbreviations

AAV: Adeno-associated viral vector
Ad5: Adenovirus type 5
ART: Antiretroviral therapy
ATL: Adult T-cell leukemia/lymphoma
ASO: Antisense oligonucleotides
BEF: Bovine HPV E2 fused to FokI
BL: Burkitt’s lymphoma
CIN: Cervical intraepithelial neoplasia
CPP: Cell penetrating peptides
crRNA: CRISPR RNA
CRISPR/Cas: The clustered regularly interspaced short palindromic repeats with CRISPR-associated protein
DSB: Double stranded break
EBNA1: EBV nuclear antigen 1
EBV: Epstein–Barr virus
HDR: Homology directed repair
HPV: Human papillomavirus
HTLV-1: Human T-cell leukemia virus type 1
HHV: Human herpesvirus
Indel: Insertion and deletions
KSHV: Kaposi’s sarcoma-associated herpesvirus
KS: Kaposi’s sarcoma
LT: Large tumor antigen
LANA: Latency-associated nuclear antigen
LMP1: Latent membrane protein 1
LCR: Long control region
LTR: Long terminal repeat
MCC: Merkel cell carcinomas
MCPyV: Merkel cell polyomavirus
MiRNA: MicroRNA
MCD: Multicentric Castleman disease
NPC: Nasopharyngeal carcinoma
NHEJ: Non-homologous DNA end joining
PEL: Primary effusion lymphoma
PAM: Protospacer adjacent motif
pRb: Retinoblastoma protein
RNAi: RNA interference
RNP: Ribonucleoprotein
RVD: Repeat-variable diresidues
sT: Small tumor antigen
TALEN: Transcription activator-like effector nuclease
TracrRNA: Trans-activating RNA
TA: Tumor antigen
vcyclin: Viral cyclin
vFLIP: Viral FLIP
ZFN: Zinc finger nuclease
